# Supplementary material for: PrEP knowledge, acceptability, and implementation in Ghana: Perspectives of HIV service providers and MSM, trans women, and gender diverse individuals living with HIV
Source: PLOS Glob Public Health. 2023 Jun 7;3(6):e0001956. doi: 10.1371/journal.pgph.0001956 (PMC10246804; doi:10.1371/journal.pgph.0001956)
Supplement: Attachment — Submitted filename: Response to reviewers_PrEP PLOS GPH.docx [file pgph.0001956.s001.docx]

**Response to reviewer comments**

Dear Dr. Carels,

Thank you for the opportunity to revise and resubmit our manuscript. We have given careful consideration to all the issues raised by the reviewers and have revised our manuscript accordingly. Below is a point-by-point clarification and explanation of revisions made in response to the reviewers. We have also slightly modified the manuscript title to reflect some of the changes that were made.

Sincerely,

Manuscript authors

**Reviewer Comments:**

**Reviewer 1**

This is a really interesting, well written and insightful article about PrEP in Ghana. I was initially concerned by the research having been conducted primarily with individuals who have HIV and were therefore not PrEP candidates, however I feel the authors addressed these concerns well and presented a compelling case for why this research matters. In addition, the introduction and methods were very strong. I have some minor comments which I think will strengthen the manuscript.

**Response**: Thank you.

**1.** I understand the framing around the sample as coming under the umbrella of MSM but given that trans women were included I do not think it appropriate that it be described as such. Instead, the authors might consider MSM (cis and trans) and gender diverse people or something similar (especially if trans men were included as well as trans women).

This is especially important given that such a high proportion have female reported for gender (n=8) and given the inclusion criteria which includes those assigned male at birth or identifying as men. This would benefit from elaboration as it isn’t clear if this is the sex (gender) assigned at birth and these individuals are trans men, or if a number of participants assigned male at birth identified as female and other as trans women.

Essentially, as it stands it is difficult to understand the sample composition and the label of MSM does not help the reader understand and therefore weakens the article.

**Response**: Thank you for raising this important point. We aimed to address this by including a statement in our methods section on the diverse gender identities shared by study participants and subsequently used gender neutral language to refer to them in the results section. Specifically, we recruited participants who were assigned male at birth who have sex with men, recognizing that this population will be gender expansive. None of our study participants identified as trans men.

Taking your suggestion, we have expanded the umbrella of MSM to “cis men, trans women, other and gender diverse individuals assigned male at birth who have sex with men” (MSM + GDSM) and have edited all references to MSM to MSM + GDSM. We have also modified the statement on gender diversity in the methods section on page 6 and have included in the statement a clarification that none of the participants identified as trans men. The manuscript title have been modified to the following to reflect the change in terminology:

**“**PrEP knowledge, acceptability, and implementation in Ghana: Perspectives of HIV service providers and MSM, trans women, and gender diverse individuals living with HIV”

**2.** It is helpful that the authors describe recruiting people who have HIV as a limitation. It is worth stating here that it is critical that further research is conducted with a range of candidates for or user of PrEP in Ghana, rather than just partners in sero-different relationships.

**Response**: We have expanded the reference to serodiscordant partners of MSM to include key populations, including trans women and other GDSM in the limitations section.

**3.** It is worth including demographic details alongside participant quotes. It is a lot of work for the reader to have to refer back to the tables to contextualise the quotes.

**Response:** We initially opted to not include the demographic details in all quoted participants due to concerns about the length of the paper. We have now added demographics (sexual identity, gender identity, and age) to the quoted MSM+GDSM clients and the roles/organization type for the quoted service providers and key informants.

**4.** Have the authors made all data underlying the findings in their manuscript fully available (please refer to the Data Availability Statement at the start of the manuscript PDF file)?

The [PLOS Data policy](https://urldefense.com/v3/__https:/journals.plos.org/globalpublichealth/s/data-availability__;!!LQC6Cpwp!sjzSI3vwmKhkTsYORK6y_jhq2cguZ15iHixW_kQCNIAmJl7d9QV4YR-h9NgIrMWt-q_hamqRdkKvQRNpP4E4WA$) requires authors to make all data underlying the findings described in their manuscript fully available without restriction, with rare exception. The data should be provided as part of the manuscript or its supporting information, or deposited to a public repository. For example, in addition to summary statistics, the data points behind means, medians and variance measures should be available. If there are restrictions on publicly sharing data—e.g. participant privacy or use of data from a third party—those must be specified.

**Reviewer #1**: No

**Response**: In our submission, we included a link to a depository of the data used for our analysis. We have posted the link below as well. We believe this depository should meet PLOS Data policy.

https://datadryad.org/stash/share/FKrj-_GS2LaWLiAwbgQ7agiA_B1yb8a10S09sdWe1k8
